# Epidemiological characteristics of mycoplasma pneumoniae in hospitalized children before, during, and after COVID-19 pandemic restrictions in Chongqing, China

**DOI:** 10.3389/fcimb.2024.1424554

**Published:** 2024-08-16

**Authors:** Jingyi You, Linghuan Zhang, Wei Chen, Qifan Wu, Dayong Zhang, Zhengxiu Luo, Zhou Fu

**Affiliations:** ^1^ Department of Respiratory Medicine, Children’s Hospital of Chongqing Medical University, National Clinical Research Center for Child Health and Disorders, Ministry of Education Key Laboratory of Child Development and Disorders, Chongqing Key Laboratory of Child Rare Diseases in Infection and Immunity, Chongqing, China; ^2^ Big Data Engineering Center, Children’s Hospital of Chongqing Medical University, Chongqing, China; ^3^ Department of Clinical Molecular Medicine, Children’s Hospital of Chongqing Medical University, Chongqing, China

**Keywords:** mycoplasma pneumoniae, children, COVID-19, epidemiology, restrictive measures

## Abstract

**Background:**

Mycoplasma pneumoniae (MP) is a significant cause of community-acquired pneumonia with high macrolide resistance rates. Various COVID-19 pandemic restrictions have impacted the prevalence of MP.

**Objective:**

To assess the changes in the pattern of MP infections among children before, during, and after the COVID-19 pandemic.

**Methods:**

A total of 36685 enrolled patients, aged 0-18 years, diagnosed with pneumonia and admitted to Children’s Hospital of Chongqing Medical University from January 2019 to December 2023, were retrospectively reviewed in this study. The epidemiological characteristics of pediatric MP infection were analyzed.

**Results:**

Among 36685 patients, 7610 (20.74%) tested positive for MP. The highest positive rate was observed among children aged over 6 years (55.06%). There was no gender disparity in MP infection across the three phases of the COVID-19 pandemic. Hospital stays were longest for children during the COVID-19 pandemic (*P* <0.001). MP infection was most prevalent in the summer (29.64%). The lowest positive rate was observed during the pandemic, with the highest rate found after easing the measures across all age groups (*P* <0.001). There was a surge in the positive rate of MP in the third year after the COVID-19 pandemic. Regression analyses demonstrated a shift in the age range susceptible to MP infection, with children aged 3.8 to 13.5 years post-pandemic compared to the pre-pandemic range of 5.3 to 15.5 years old. Additionally, the average macrolide resistance rate was 79.84%. We observed a higher resistance rate during the pandemic than in the pre- and post-pandemic phases (*P* <0.001).

**Conclusion:**

The restrictive measures implemented during the COVID-19 pandemic have influenced the spread of MP to some extent and altered demographic and clinical characteristics, such as age, age group, season, length of stay, and macrolide resistance. We recommend continuous surveillance of the evolving epidemiological characteristics of MP infection in the post-pandemic period when restrictions are no longer necessary.

## 1 Introduction

Mycoplasma pneumoniae (MP) is a prevalent pathogen responsible for community-acquired pneumonia (CAP) worldwide (Parrott et al., 2016). Mycoplasma pneumoniae pneumonia (MPP) constitutes 10-40% of CAP hospitalizations among children, particularly among school-aged children and adolescents (Gao et al., 2019; Guo et al., 2022). Clinical manifestations of MPP vary, and it can develop into severe life-threatening complications such as necrotizing pneumonia, plastic bronchitis, and respiratory failure (Zhang et al., 2021). Macrolides are the primary treatment for MP infections in children (Tsai et al., 2021). However, the high prevalence of macrolide resistance in Asia could lead to poor clinical outcomes (Zhang et al., 2024). Consequently, children are susceptible to MP infection, with significant morbidity and mortality.

MP is a self-replicating prokaryotic microorganism with a highly stable genome (0.8Mbp) that lacks a cell wall and exhibits slow growth (generation time 6 hours) (Meyer Sauteur and Beeton, 2023a; Reimann, 1984). It is commonly genotyped using P1 typing and multiple-locus variable-number tandem-repeat analysis (MLVA). P1 gene typing comprises two main subtypes, namely typ1 and typ2, while MLVA typing has identified five variable-number tandem-repeat loci (Mpn1 and Mpn13-16) (Kenri et al., 2008; Dégrange et al., 2009). MP is primarily transmitted through air droplets and close contact. The incubation period is 1-3 weeks, and it remains contagious from the incubation period to several weeks after clinical symptom relief (Yan et al., 2024).

The infection occurs in any season worldwide, with epidemic peaks occurring every few years (Uldum et al., 2012). In northern China, MP infections predominantly occur in autumn and winter, while in southern China, transmission usually peaks in the summer and autumn (Wang et al., 2022). Previous data indicated that the interval between MP epidemics in Europe and Israel is 1-3 years (Beeton et al., 2020), with each outbreak lasting for 1-2 years (Lenglet et al., 2012). The most recent epidemic of MP occurred from late 2019 to early 2020 across multiple countries (Meyer Sauteur and Beeton, 2023a). Since the outbreak of COVID-19 in December 2019 in Wuhan, nonpharmaceutical interventions (NPIs), including the wearing of masks, hand-sanitizing, and social distancing, have been implemented to control the spread of severe acute respiratory syndrome coronavirus 2 (SARS-CoV-2). These efforts have also resulted in a significant decline in MP detection worldwide and a sudden ending of these epidemics (Zhang et al., 2022).

Similar to other respiratory pathogens, the incidence of MP showed a significant decrease in the first year following the implementation of NPIs, which dropped from 8.61% (2017-2020) pre-pandemic to 1.69% (2020-2021) (Meyer Sauteur et al., 2022a). When other respiratory pathogens experienced a resurgence in the second year, a further decline in the incidence of MP (0.70%, 2021-2022) was observed (Meyer Sauteur et al., 2022b). This low incidence (0.82%) persisted into the third year post-pandemic (April 2022 to March 2023) (Meyer Sauteur and Beeton, 2023b). After three years of low detection rates, a marked increase in the incidence of MP was noted in children (Bolluyt et al., 2024; Gong et al., 2024; Zhang et al., 2024). The resurgence in MP-infected children has attracted widespread attention to this epidemic.

Data on the epidemiological characteristics of MP at different stages of the COVID-19 pandemic in China over a long period is limited. In this five-year retrospective study, we aimed to investigate the prevalence of MP infection among hospitalized children in Chongqing before, during, and after the COVID-19 pandemic from January 1, 2019, to December 31, 2023.

## 2 Methods

### 2.1 Study design

Patients diagnosed with pneumonia and admitted to the respiratory ward of the Children’s Hospital of Chongqing Medical University (CHCMU) between January 1, 2019, and December 31, 2023, were enrolled in this retrospective study. CHCMU is the largest children’s hospital in southwestern China. The inclusion criteria were as follows: 1) children under the age of 18 years hospitalized for pneumonia; 2) available results of real-time PCR assay for MP. Patients were excluded if they met any of the following conditions: 1) children with severe malformations, such as congenital heart disease, immune deficiency, or chronic lung disease; 2) children diagnosed with malignant tumors or who used immunosuppressive drugs during hospitalizations.

All enrolled children were divided into four groups based on their age: (1) 0-12 months; (2) 1-3 years; (3) 3-6 years; (4) over 6 years old. Seasons were divided into four groups, that is, spring (March-May), summer (June-August), autumn (September-November), and winter (December-February). The study period was divided into three phases based on the management of the COVID-19 pandemic in China: (1) Phase I: before the COVID-19 pandemic, spanning from January 1, 2019, to January 23, 2020. (2) Phase II: during the COVID-19 pandemic, covering the period from January 24, 2020, to December 11, 2022. (3) Phase III: post-COVID-19 pandemic, starting after December 11, 2022, when Chongqing announced the complete relaxation of COVID-19 restrictive measures.

### 2.2 Specimens collection and detection

Nasopharyngeal aspirate or bronchoalveolar lavage (BAL) fluid from the enrolled children was collected by trained medical staff and immediately transported to the Clinical Molecular Laboratory Center. BAL was performed by locating the affected lobes and segments using flexible bronchoscopes (Olympus) and chest imaging. A sterile saline solution at a temperature of 37°C was instilled at a volume of 1ml/kg (not exceeding 20ml), then the fluid of each portion was recovered by gentle aspiration. A total of 5 to 10mgl/kg (depending on the physician’s choice) was instilled in each patient. A 10ml sample of BAL was collected and detected using polymerase chain reaction (PCR) and melting curve method in strict accordance with the diagnostic kit (DaAn Gene Co., Ltd. Guangzhou, China). Primers and probes targeted to 16s rRNA were used for MP detection. Detection of macrolide-resistant mutation sites (23S rRNA 2603 (A: G) and 2064 (A: G)) for MP was performed using a kit (MoLe Biotech Co., Ltd. Jiangsu, China) with PCR fluorescence probing technology. Both methods are based on the TaqMan PCR technology and were conducted by professional staff following standard operating procedures as previously reported (He et al., 2013).

### 2.3 Data collection

Demographic and clinical data, such as age, gender, admission date, length of stay, and MP DNA detection results (positive/negative), were obtained from the Big Data Engineering Center. Macrolide-resistant Mycoplasma pneunomiae (MRMP) detection results (positive/negative)) were extracted from the Clinical Molecular Medicine Laboratory Information System. The MRMP testing began on April 1, 2019, at the Clinical Molecular Medicine Center.

### 2.4 Statistical analysis

Data extracted from January 1, 2019, to December 31, 2023, were analyzed using SPSS version 29.0 software (IBM Co., Armonk, NY, USA). The positive detection rates of MP and their 95% confidential interval (CI) were calculated for all patients and patients of different ages across subgroups categorized by gender, season, and the three phases of the COVID-19 pandemic. Continuous variables, including length of stay and age, were described as the median and interquartile range (IQR) [M (P_25_, P_75_)]. In contrast, categorical variables, including gender, age group, and season, were described as numbers and percentages. Comparisons of continuous variables were performed using the Kruskal-Wallis rank test, while categorical variables were analyzed using the Chi-squared test. *P*-value (two-tailed) <0.05 was considered statistically significant.

The nonlinear association between age and positive detection rate across different pandemic phases was analyzed using the restricted cubic spline regression model in software R version 4.3.3 (R Foundation for Statistical Computing, Vienna, Austria). We chose four knots for the RCS model, positioned at the 5th, 35th, 65th, and 95th percentiles.

## 3 Results

### 3.1 Positive detection rates of MP across age groups

A comprehensive analysis was conducted on a total of 36685 children, aged 0 to 18 years, at the CHCMU (**Table 1**). The children were categorized into four age groups: 14473 infants (0-12 months), 9212 toddlers (1-3 years), 7510 preschool children (3-6 years), and 5490 school-age children and adolescents (over 6 years). The lowest positive rate was observed in patients aged 0-12 months (5.38% (5.01%-5.74%)), which gradually increased with age, peaking at the highest positive rate in patients over 6 years old (55.06% (53.75%-56.38%)). Male patients younger than 1 year old had a statistically significantly higher positive rate (5.98% (5.50%-6.45%))(*P* <0.001). In contrast, male patients aged 1-3 years old (15.00% (14.08%-15.92%)) and over 6 years old (52.08% (50.20%-53.96%)) had a statistically significantly lower positive rate (*P* =0.012 and *P* <0.001, respectively). The highest positive rates for most age groups were found in summer (i.e., 9.32% (8.30%-10.35%) for 0-12 months, 24.17% (22.47%-25.87%) for 1-3 years old, 40.15% (38.05%-42.26%) for 3-6 years old), except for patients over 6 years old, who had the highest positive rate in autumn (61.42% (58.98%-63.86%)). Significant differences were observed in the positive rates across the three phases at different age groups. Compared to Phase I, the positive rates significantly decreased in Phase II, but increased in Phase III compared to both Phase I and Phase II (*P* <0.001).

**Table 1:** Positive detection rates and 95% confidence intervals of MP infection across age groups.

Variables	All patients	0-12 months	1-3 years	3-6 years	≥ 6 years
Number	36685	14473	9212	7510	5490
MP positive number	7610	778	1449	2360	3023
MP (%)	20.74 (20.33-21.16)	5.38 (5.01-5.74)	15.73 (14.99-16.47)	31.43 (30.38-32.48)	55.06 (53.75-56.38)
Gender (%)					
Male	18.51 (17.99-19.02)	5.98 (5.50-6.45)	15.00 (14.08-15.92)	30.89 (29.40-32.39)	52.08 (50.20-53.96)
Female	24.09 (23.40-24.78)	4.57 (3.98-5.16)	16.96 (15.71-18.22)	31.93 (30.46-33.40)	57.99 (56.15-59.83)
*P*-Value	<0.001	<0.001	0.012	0.333	<0.001
Season (%)					
Spring	10.05 (9.43-10.68)	2.72 (2.21-3.22)	7.18 (6.09-8.27)	16.39 (14.67-18.10)	35.72 (32.67-38.78)
Summer	29.64 (28.72-30.56)	9.32 (8.30-10.35)	24.17 (22.47-25.87)	40.15 (38.05-42.26)	58.88 (56.64-61.12)
Autumn	26.10 (25.22-26.98)	7.09 (6.22-7.97)	19.52 (17.97-21.07)	37.31 (35.30-39.33)	61.42 (58.98-63.86)
Winter	16.16 (15.39-16.93)	3.62 (3.05-4.20)	10.19 (8.90-11.49)	28.32 (25.97-30.67)	56.40 (53.54-59.26)
*P*-Value	<0.001	0.196	<0.001	<0.001	<0.001
Pandemic phases (%)					
Phase I	22.39 (21.52-23.26)	7.10 (6.29-7.90)	21.65 (19.96-23.33)	41.45 (39.05-43.84)	56.81 (53.64-59.98)
Phase II	11.54 (11.09-12.00)	2.31 (1.98-2.64)	8.09 (7.33-8.85)	18.23 (16.99-19.47)	40.17 (38.18-42.16)
Phase III	39.98 (37.95-40.00)	12.65 (11.34-13.96)	28.41 (26.39-30.43)	47.01 (44.89-49.14)	70.02 (68.11-71.93)
*P*-Value	<0.001	<0.001	<0.001	<0.001	<0.001

Data are presented as absolute frequency and percentage (95% CI). The Chi-squared test was applied for comparison. MP positive number: the number of hospitalized children with pneumonia who tested positive for mycoplasma pneumoniae.

### 3.2 Demographic and clinical characteristics of MP infection before, during, and after COVID-19 pandemic restrictions

The analysis of demographic and clinical characteristics of cases with MPP between 2019 and 2023 was presented (**Table 2**). A total of 7610 children were admitted for analysis, including 1986 (26.10%) in Phase I, 2212 (29.07%) in Phase II, and 3412 (44.83%) in Phase III. The median length of stay for children diagnosed with MPP across the three phases was 6 days, and the median age was 5 years. Among the total cases, 4069 (53.47%) were male, 3023 (39.72%) were older than 6 years, and 2806 (36.87%) were tested in summer. Statistically significant differences were observed among the three pandemic phases in terms of length of stay, age, age group, and season (all *P*-values <0.001). There was longer median (IQR) length of stay in Phase II (6.0 (5.0-8.0)) compared to Phase I (6.0 (4.0-7.0)) and Phase III (5.0 (4.0-8.0)). The median (IQR) age in Phase I (3.8 (1.8-6.2)) was lower than in Phase II (5.3 (2.8-7.6)) and in Phase III (5.6 (3.0-7.5)). In all three phases, more than half were boys. The largest age group in Phase II (42.48%) and Phase III (45.41%) was children older than 6 years old, while in Phase I, it was children between 3 and 6 years old (34.04%). Most cases were observed in summer and autumn, with positive rates approximately reaching 70%.

**Table 2:** Demographic and clinical characteristics of MPP before, during, and after COVID-19 pandemic restrictions.

Characteristics	Overall	Phase I	Phase II	Phase III	*P*-Value
Number	7610	1986	2212	3412	
length of stay	6.0 (4.0-8.0)	6.0 (4.0-7.0)	6.0 (5.0-8.0)	5.0 (4.0-8.0)	<0.001
Age (years)	5.0 (2.5-7.3)	3.8 (1.8-6.2)	5.3 (2.8-7.6)	5.6 (3.0-7.5)	<0.001
Gender					
Male	4069 (0.53)	1109 (0.56)	1167 (0.53)	1793 (0.53)	0.050
Female	3541 (0.47)	877 (0.44)	1045 (0.47)	1619 (0.47)	
Age group					<0.001
0-12 months	778 (0.10)	276 (0.14)	187 (0.08)	315 (0.09)	
1-3 years	1449 (0.19)	500 (0.25)	403 (0.18)	546 (0.16)	
3-6 years	2360 (0.31)	676 (0.34)	684 (0.31)	1000 (0.29)	
≥6 years	3023 (0.40)	534 (0.27)	938 (0.42)	1551 (0.45)	
Season					<0.001
Spring	896 (0.12)	257 (0.13)	412 (0.19)	227 (0.07)	
Summer	2806 (0.37)	837 (0.42)	927 (0.42)	1042 (0.31)	
Autumn	2493 (0.33)	567 (0.29)	493 (0.22)	1433 (0.42)	
Winter	1415 (0.19)	325 (0.16)	380 (0.17)	710 (0.21)	

Data are presented as median, interquartile range, absolute frequency, and percentages. The Kruskal-Wallis test for continuous variables and the Chi-squared test for categorical variables were used for comparison.

### 3.3 Monthly and seasonal changes in MP infection in children from 2019 to 2023

**Figure 1:**
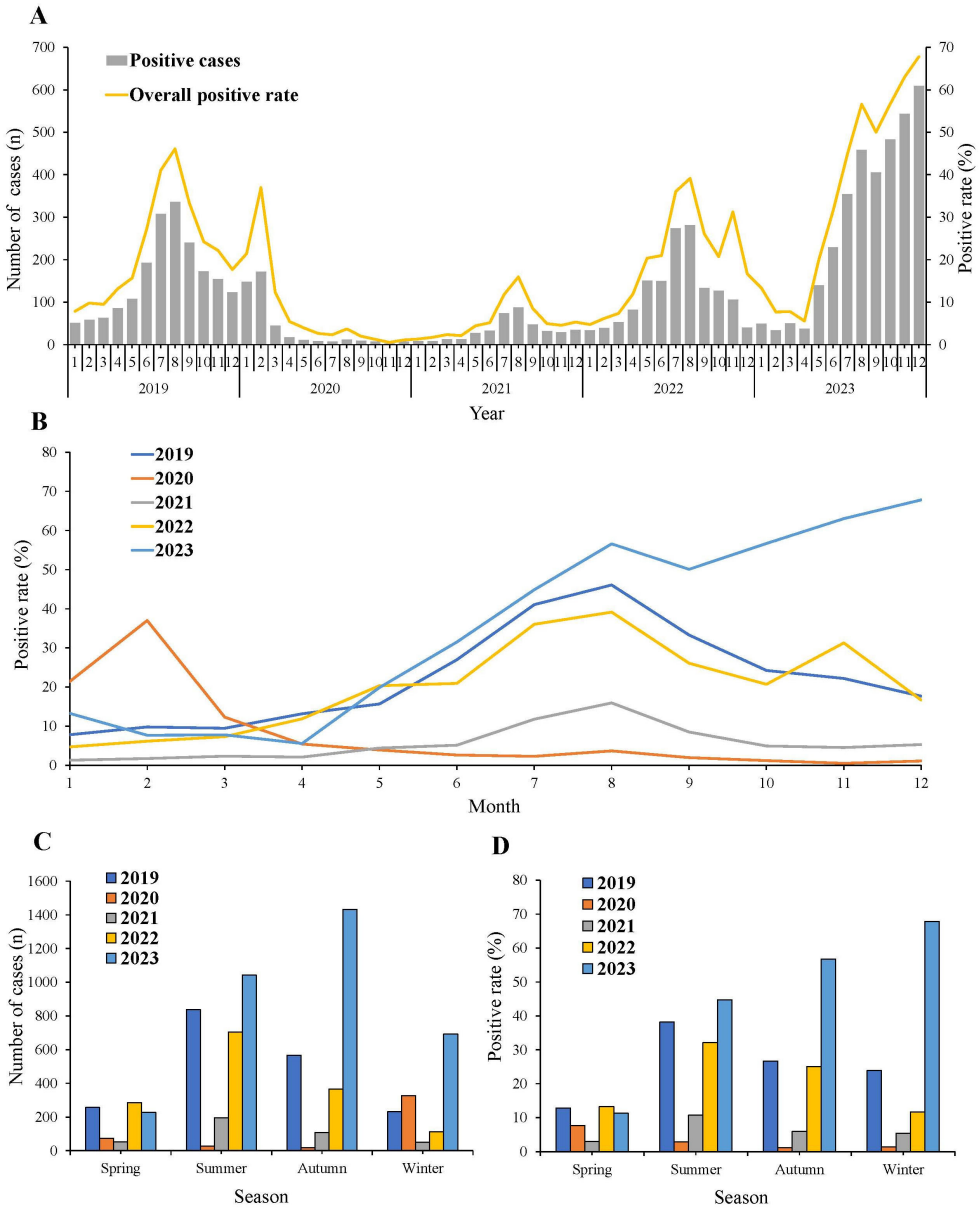
Monthly and seasonal distribution of MP infection from 2019 to 2023. **(A)** General trend of MP positive rate and number from 2019 to 2023. **(B)** MP positive rate in each month in each year. **(C)** MP positive number and **(D)** positive rate in different seasons.

In 2019, the number and positive rate of MP exhibited monthly fluctuations. However, during the initial two years of the COVID-19 pandemic, both metrics experienced a dramatic decrease from April 2020 to February 2022. Despite an increase in both the number and positive rate of MP during the third year of the COVID-19 pandemic, they remained lower than pre-pandemic levels. The detection rate of MP began to surge in April 2023, followed by a slight decrease in September. However, it continued to rise, reaching as high as 67.8%. This surge significantly exceeded the levels observed before the COVID-19 pandemic. Notably, in the last five months of 2023, more than 50% of hospitalized children with pneumonia in our respiratory ward were diagnosed with MPP (**Figure 1A**).

In 2019, the positive rate of MP started to rise from March, reaching its peak in August, and then began to decline. In 2020, the MP positive rate peaked in February, after which it sharply decreased and remained at notably low levels. Throughout 2021, the incidence of MP remained consistently low. The trend of MP positive rate in 2022 was similar to that of 2019 and 2021, with rates falling between the two. Prior to August 2023, the trend of the MP positive rate was similar to that of 2019, 2021, and 2022. However, post-August 2023, in contrast to the downward trend observed in the previous three years, an upward trend was noted (**Figure 1B**).

The seasonal distribution of the number and positive rates of MP was analyzed (**Figures 1C, D**). The positive rates of MP decreased across all seasons in 2020 and 2021. Throughout 2019, 2021, and 2022, the number and positive rate of MP exhibited a consistent pattern. They all peaked in summer and then started to decline in autumn, remaining at low levels during winter and spring. In 2020, the positive rate was highest in spring, while in winter 2023, it surpassed rates observed in other seasons.

### 3.4 Nonlinear associations between age and positive rate of MP

**Figure 2:**
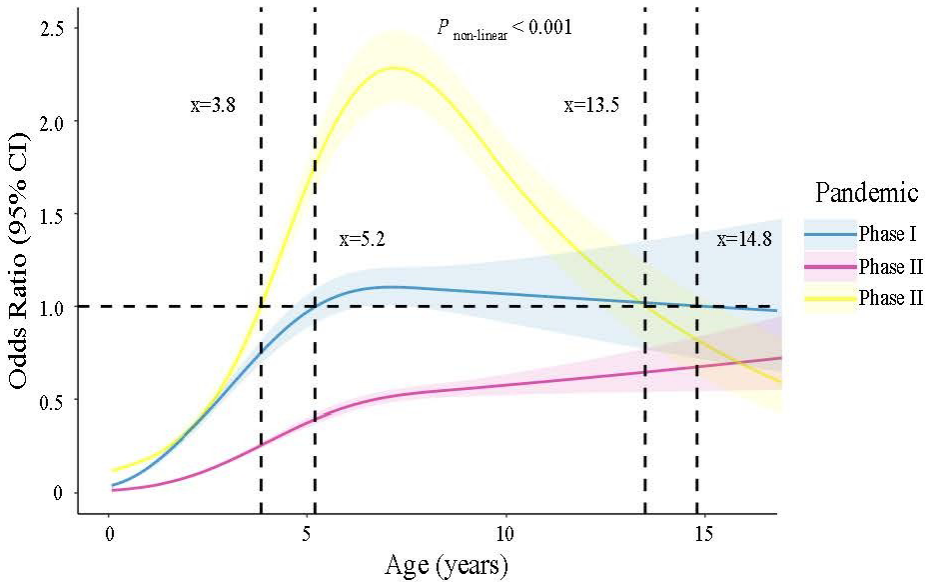
Nonlinear association between age and risk of MP infection estimated by the restricted cubic spline regression model.

The nonlinear relationship between age and the risk of MP infection across three different phases of the COVID-19 pandemic was investigated using restricted cubic spine regression models (*P_nonlinear_* <0.001) (**Figure 2**). In phase I, patients aged 5.2 to 14.8 years old had odds ratios (ORs) >1. In Phase II, all patients had ORs <1, indicating a lower risk of MP infection compared to Phase I and Phase III. In Phase III, the age range with ORs >1 shifted from 3.8 to 13.5 years old compared to Phase I. Additionally, among patients approximately under 13.5 years old, the highest OR values were observed in Phase III.

### 3.5 Changes of macrolide resistance in MP before, during, and after the COVID-19 pandemic

**Figure 3:**
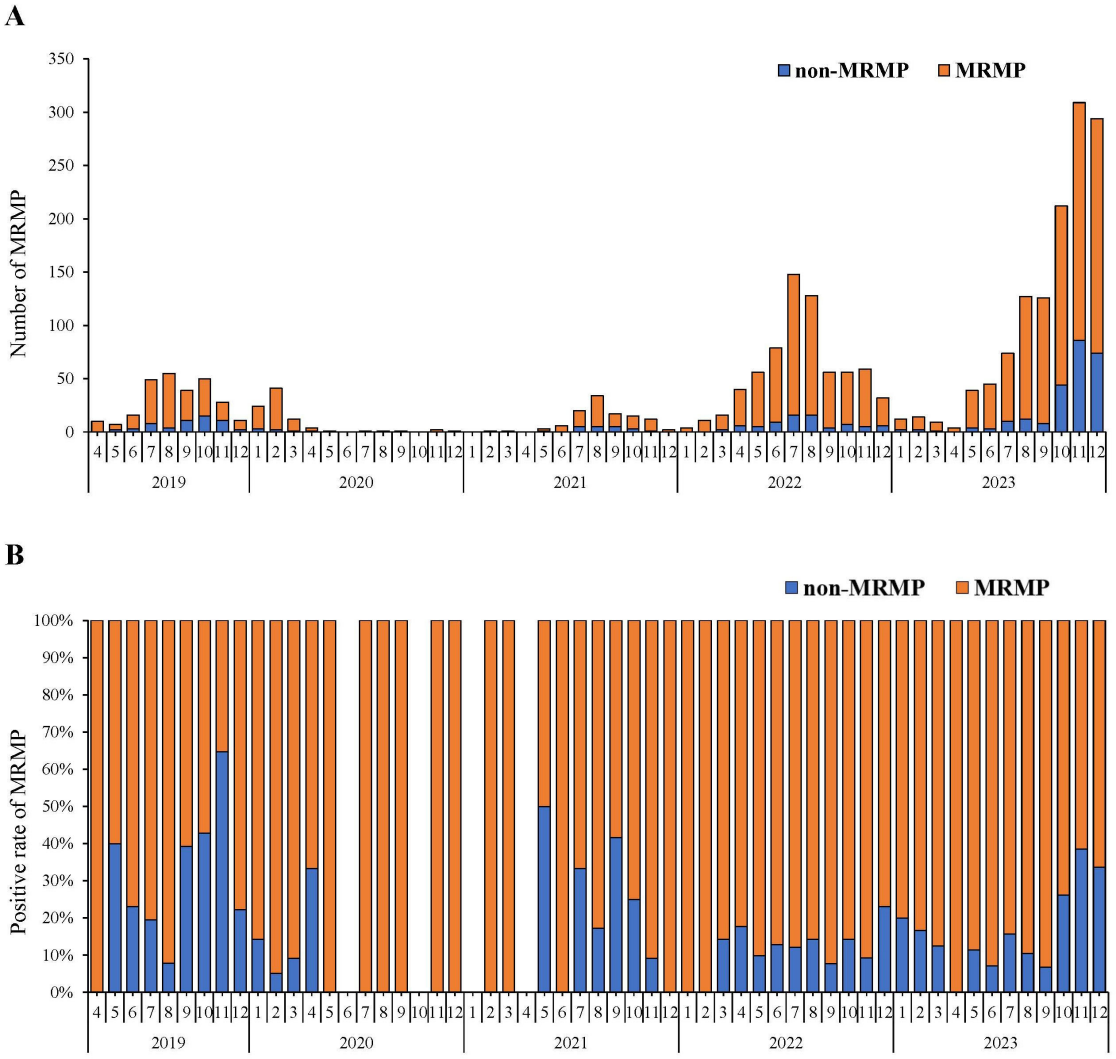
Macrolide-resistant Mycoplasma Pneumoniae (MRMP) testing and detection from April 2019- December 2023. **(A)** Numbers of MRMP positives in different months. The red parts of the bar graph represent the absolute numbers of MRMP detection. **(B)** Percentages of MRMP positive rate in different months. The red parts of the bar graph represent the proportions of MRMP detection.

From April 2019 to December 2023, a total of 2009 children underwent testing for macrolide resistance in mycoplasma pneumoniae (MRMP). The MRMP detections among these children are shown as absolute numbers (**Figure 3A**) and as percentages (**Figure 3B**). The trend in the number of individuals undergoing MRMP testing corresponds to that of MP infection (**Figure 1A**). Over the 5-year period, the MRMP positive rate remained consistently above 50% except for November 2019. The overall average positive rate was 79.84% (1604/2009). The MRMP positive rate was significantly higher in Phase II, at 87.07% (660/758) compared to 73.87% (164/222) in Phase I and 75.80% (780/1029) in Phase III (*χ* ^2^ =38.991, *P* <0.001).

## 4 Discussion

Chongqing, located in southwest China, is home to a population of over 32 million people. After the outbreak of COVID-19, Chongqing implemented stringent NPIs from January 23, 2020, to control the spread of SARS-CoV-2. All restrictive measures were lifted on December 11, 2022. Similar to SARS-CoV-2, MP is transmitted through the respiratory tract, and the NPIs enacted for COVID-19 also contributed to the reduction in MP transmission (Cheng et al., 2022; Ma et al., 2023; Meyer Sauteur et al., 2022a). In our study, we observed a decrease in the positive detection rate of MP across all age groups during the pandemic compared to pre-pandemic levels. However, after the lifting of restrictions, there was a subsequent increase in detection rates. It is reasonable to attribute the higher detection rate of MP after the lifting of restrictive measures compared to the pre-pandemic period to the phenomenon known as immunity debt. The long-term implementation of NPIs in Chongqing resulted in a prolonged lack of exposure to the pathogen among the population, coupled with the absence of a vaccine, thereby leading to a decrease in immune levels and an increase in susceptible populations.

During the COVID-19 pandemic, we observed prolonged hospital stays for children compared to pre- and post-pandemic phases. This may be related to more severe illness, a higher co-infection rate, and potential selection bias. Previous studies have suggested that variations in genotype could impact different clinical manifestations and the occurrence of SMPP (Yan et al., 2019; Li et al., 2022). Additionally, the choice of mild MPP patients to seek treatment at a nearby hospital due to the lockdown measures could impact the duration of hospital stays. The relaxation of pandemic-related measures led to a resurgence of other respiratory pathogens, such as respiratory viruses (RSV) and influenza (Clark et al., 2023; Meyer Sauteur et al., 2022b), potentially contributing to longer hospital stays through co-infection with MPP. However, clinical symptoms and outcomes of other pathogenic infections were not included in our study, so we cannot pinpoint the specific reasons for prolonged hospital stays during the pandemic. Further research incorporating relevant data is necessary to address this gap.

According to our study, children over 3 years old, especially those over 6 years old, had a relatively higher risk of MP infection throughout all phases of the COVID-19 pandemic. This finding is consistent with previous reports from several European countries (Chalker et al., 2012), the United States (Kutty et al., 2019), and India (Kumar et al., 2018). School-aged children are more susceptible to MP infection due to their increased exposure to pathogens in daily activities compared to infants and toddlers, which increases the risk of infection (Ma et al., 2023). Meanwhile, the relatively mature immune system of school-aged children can lead to inflammatory responses when MP infects the respiratory tract. This may cause immune damage to respiratory tissues, providing favorable conditions for the growth and reproduction of the pathogen (Zhu et al., 2023). Furthermore, the loss of immune mediators acquired from the mother at this age also contributes to the high incidence of MP infection (Hill et al., 2020). Our analysis of gender differences in MP infections revealed no disparity between boys and girls across all phases of the COVID-19 pandemic, which corresponds with previous findings (Waites et al., 2019; Ma et al., 2023; Zhang et al., 2021).

MP infection is not restricted to any particular season and exhibits variation in epidemic patterns across different regions of China (Wang et al., 2022; Zhang et al., 2024). The optimal growth temperature for MP ranges between 35°C and 37°C, suggesting that warmer climates could prolong its environmental survival and facilitate broader dissemination. Consistent with previous studies (Yan et al., 2024), our findings suggested that MP infections occur throughout the year in Chongqing, located in southwest China, with a particular prevalence in summer. Except for 2020, the positive rate of MP increased from early spring (March), peaking in late summer (August). However, in 2020, the seasonal pattern of MP was disrupted due to restrictive measures such as home isolation, travel bans, temporary closures of public places, regular hand sanitizing, and mask-wearing.

A global prospective surveillance showed a resurgence of MP occurred in the fourth year following the COVID-19 outbreak (Meyer Sauteur and Beeton, 2024). This finding corresponds to a report that summarized data from 2017 to 2023 in eastern China, indicating that the incidence of MP ranged from 10% to 20% during the COVID-19 period (2020-22) (Li et al., 2024). This delayed re-emergence is atypical and potentially unique to MP. The slower generation time (6 h), longer incubation period (1-3 weeks), and lower transmission rate of MP may require a longer time interval for the re-establishment of MP infection within a population (Meyer Sauteur et al., 2022b). Variations in MP subtypes could also contribute to epidemiological changes (Li et al., 2022). Intriguingly, our study observed an increase in the positive rate of MP occurred in the third year following the COVID-19 pandemic, starting from May and ranging from 20.4%-39.7%, with another surge beginning in the fourth year in August, exceeding 50%, and reaching as high as 67.8% in December. The disparity in these results may be attributed to geographical factors favoring MP proliferation in the hot climate of Chongqing. We recommend replicating this study in other populations to uncover the epidemiological characteristics of MP.

Our study investigated the nonlinear relationship between age and the risk of having MP infection across three phases of the COVID-19 pandemic. We found a general decline in MP susceptibility among the population during the pandemic. However, there was a notable increase in the risk of MP infection following the relaxation of restrictions. Moreover, our data demonstrated a shift in the age range of children susceptible to MP, with the range shifting from 5.3 to 15.5 years old before the pandemic to 3.8 to 13.5 years old after the pandemic, indicating a shift of approximately 1.5 to 2.0 years towards younger age. This shift implies that younger children may become vulnerable to MP after easing restrictions. These findings have significant implications for both public health strategies and clinical practice.

Macrolide antibiotics are recommended as the primary treatment option for MP infection. However, their widespread use has led to the emergence of treatment resistance (Tsai et al., 2021). To our knowledge, this is the first large-scale study conducted in China to investigate macrolide resistance across three phases of the COVID-19 pandemic. Our findings revealed an average resistance rate of 79.84% from 2019 to 2023. This result corresponds with research conducted in Asian countries, including China, Japan, and South Korea, which reported resistance rates ranging from 80% to 90% between 2013 and 2019 (Yamazaki and Kenri, 2016; Chen et al., 2020; Leng et al., 2023). The high prevalence of MRMP may be correlated with genotype shifting and macrolide usage. Previous reports have indicated that macrolide resistance is associated with the specific genotype P1 type 2 and MLVA type M4-5-7-2 (Zhao et al., 2013; Yan et al., 2015). Multiple hospitals have observed that the outbreak of MP infection in 2023 is predominantly caused by MRMP strains (Yan et al., 2024; Zhang et al., 2024). However, our study uncovered a higher resistance rate during the pandemic compared to the pre-pandemic and post-pandemic phases. This increase in resistance may be related to potential selection bias due to lower sample submissions during the pandemic. Given the significant burden imposed by macrolide resistance on the treatment of MP infection, epidemiological surveillance, including macrolide resistance testing of MP, is crucial for infectious disease monitoring.

This study possesses several strengths. Firstly, we benefitted from a large-scale sample comprising 36685 hospitalized children, enabling us to draw reliable conclusions. Secondly, we utilized restricted cubic spline regression analysis to assess the nonlinear relationship between age and the risk of MP infection across three pandemic phases. Thirdly, we investigated macrolide resistance patterns throughout three pandemic phases in China, which was not addressed in previous studies. Despite these strengths, our study does have several limitations. Firstly, it was carried out at a single center, and data were only collected exclusively from hospitalized patients diagnosed with pneumonia, potentially introducing selection bias and limiting the generalizability of our results. Future multicenter research is essential to formulate comprehensive control, prevention, and treatment strategies for MP infections. Secondly, the proportion of hospitalized patients over 14 years old in our study was relatively small, with 18 adolescents in Phase I, 60 adolescents in Phase II, and 51 adolescents in Phase III. This limited sample size may restrict the statistical power of our analysis. In addition, we did not include the clinical symptoms and outcomes of other pathogenic infections. Future studies involving a larger sample size with the adolescent age group and other pathogenic infections could provide additional epidemiological insights. Lastly, our study lacked data on the genetic types of MP, highlighting the necessity for further investigation into MP genome evolution.

## 5 Conclusions

In this study, we enrolled more than 35000 hospitalized children diagnosed with pneumonia over a five-year period spanning different phases of the COVID-19 pandemic to investigate the prevalence of MP infection. Our findings indicated a significant impact of restrictive measures on the prevalence and seasonal pattern of MP. Children over 6 years old were most susceptible to infection, with a forward shift in the age range of susceptible children observed when restrictions were fully lifted. Additionally, during the pandemic, both the duration of hospital stays and the rate of macrolide resistance peaked across the three phases. Based on our findings, we recommend continuous surveillance of the epidemiological characteristics of MP infection in the post-pandemic period.

## Data Availability

The original contributions presented in the study are included in the article/supplementary material. Further inquiries can be directed to the corresponding author.
